# Let Not the Cobbler Go beyond His Last

**Published:** 1885-02

**Authors:** 


### EDITORIAL.

"LET NOT THE COBBLER GO BEYOND HIS LAST."

One of Chicago's most celebrated divines has recently made his *début* as a medical writer. He has selected as the medium, through which his pathological lucubrations shall meet the public ken, a weekly magazine, devoted to "Literature, Government, Science, and Art."

With characteristic modesty, he has chosen "Cholera and the Nervous System" as his theme. The selection of this particular magazine is judicious, since its contributors are elected exclusively from the ranks of the *illuminati.* Lord Chief Justice Coleridge has recently addressed an autograph letter to one of the chief editorial writers on the staff. Coleridge has read, "constantly," "week by week," "with interest and admiration," this paper. Then the topic is peculiarly felicitous. The subject is of vital importance. Koch, Weigert, Pasteur, Klein, Klebs, Perls, v. Recklinghausen, Birch-Hirschfeld, Virchow have studied the pathology and and therapy of this strange disease for years. Up to the present time, however, the etiology, to say the least, is obscure, and treatment is not always successful. Our esteemed contemporary has arrived at clear, distinct, and adequate knowledge, both in regard to the morbid processes as well as the curative powers of certain remedial agents.

After a brief exposition of that method of medication, termed the "metaphysical treatment," the writer alludes to the theory of cholera, propounded by Dr. John Chapman, of London. "The base of the brain becomes disordered by heat or by some unknown influence in the atmosphere, and from this abnormal action of the spinal cord, the secretions become so redoubled that the fluids which support life are carried away as with a flood." "The disease is no more contagious than is ague or dyspepsia." "If the disease finds you, go to bed at once, and let the helping hands, if there are any, apply a long, narrow bag of ice to your abnormal and mischief-making spinal column, and perhaps you will live to swell the much needed *data* as to the best method of treating cholera."

We are told that Apelles, after the completion of a picture, was accustomed to expose it to the view of passers-by, hiding himself meanwhile in the vicinity, in order to hear the criticisms of the spectators. One day a shoemaker came along and criticized the number of ties upon the slipper of the painted figure. Apelles, recognizing the justness of the criticism, corrected the error. Emboldened by his success, the cobbler, on the succeeding day, passed certain strictures upon the contour of the leg. The indignant Apelles immediately put forth his head and requested the cobbler to confine his attention to the slipper. Hence arose the expression: "*Ne sutor ultra crepidam.*" "Let not the cobbler go beyond his last."

